# Ex Vivo Colonic Fermentation of NUTRIOSE^®^ Exerts Immuno-Modulatory Properties and Strong Anti-Inflammatory Effects

**DOI:** 10.3390/nu15194229

**Published:** 2023-09-30

**Authors:** Caroline Perreau, Clementine Thabuis, Lynn Verstrepen, Jonas Ghyselinck, Massimo Marzorati

**Affiliations:** 1 Nutrition and Health R&D, Roquette, 1 rue de la Haute Loge, 62136 Lestrem, France; caroline.perreau@roquette.com (C.P.); clementine.thabuis@roquette.com (C.T.); 2 ProDigest, Technologiepark 82, 9052 Zwijnaarde, Belgium; lynn.verstrepen@prodigest.eu (L.V.); jonas.ghyselinck@prodigest.eu (J.G.); 3 Center for Microbial Ecology and Technology (CMET), Faculty of Bioscience Engineering, Ghent University, Coupure Links 653, 9000 Ghent, Belgium

**Keywords:** anti-inflammatory, Colon-on-a-plate, immunomodulation, prebiotic, resistant dextrin, short-chain fatty acid

## Abstract

NUTRIOSE^®^ (Roquette, Lestrem, France) is a resistant dextrin with well-established prebiotic effects. This study evaluated the indirect effects of pre-digested NUTRIOSE^®^ on host immune response and gut barrier integrity. Fecal samples from eight healthy donors were inoculated in a Colon-on-a-plate^®^ system (ProDigest, Ghent, Belgium) with or without NUTRIOSE^®^ supplementation. Following 48 h fermentation, colonic suspensions were tested in a Caco-2/THP1-Blue™ co-culture system to determine their effects on gut barrier activity (transepithelial electrical resistance) and immune response following lipopolysaccharide stimulation. Additionally, changes in short-chain fatty acid levels (SCFA) and microbial community composition following a 48 h fermentation in the Colon-on-a-plate^®^ system were measured. Across all donors, immune-mediated intestinal barrier damage was significantly reduced with NUTRIOSE^®^-supplemented colonic suspensions versus blank. Additionally, IL-6 and IL-10 levels were significantly increased, and the level of the neutrophil chemoattractant IL-8 was significantly decreased with NUTRIOSE^®^-supplemented colonic suspensions versus blank in the co-culture models following lipopolysaccharide stimulation. These beneficial effects of NUTRIOSE^®^ supplementation were likely due to increased acetate and propionate levels and the enrichment of SCFA-producing bacteria. NUTRIOSE^®^ was well fermented by the colonic bacteria of all eight donors and had protective effects on inflammation-induced disruption of the intestinal epithelial barrier and strong anti-inflammatory effects.

## 1. Introduction

It is well-recognized that the gut microbiome has a strong influence on human health and disease. Gut microbiome dysbiosis may lead to increased bowel permeability (i.e., ‘leaky gut’) and an increase in lipopolysaccharides (LPS) and inflammatory molecules [1]. Dysbiosis is associated with several immune-related diseases [2,3,4,5] and metabolic disorders [6,7,8]. Thus, much work has been carried out to understand how to achieve and maintain a healthy gut microbiome. One approach to this is dietary supplementation with probiotics and/or prebiotics. Prebiotics are defined as substrates that are “selectively utilized by host microorganisms conferring a health benefit” [9]. These nutrients pass through the small intestine poorly digested or undigested. Upon entering the large intestine, the prebiotic fibers are fermented by gut microbes, resulting in partial or complete digestion. Prebiotics are associated with several health benefits, including beneficial changes to the gut microbiome composition and function, improvements in immune function, and increased levels of short-chain fatty acids (SCFAs) (reviewed in Devani-Davari et al. [10]). SCFAs, including acetate, propionate, and butyrate, are a major product of prebiotic fermentation [10,11] and provide many benefits to the host, including reduced intestinal inflammation and improved intestinal barrier integrity [12]. 

NUTRIOSE^®^ is a soluble fiber with prebiotic properties [13]. Approximately 15% of consumed NUTRIOSE^®^ is enzymatically digested in the small intestine, 75% is progressively fermented in the colon and the remaining 10% is excreted [13,14,15]. NUTRIOSE^®^ supplementation increases SCFA production in rats and in humans [16] and exerts beneficial changes in the gut microbiota composition of healthy human volunteers [17,18,19]. Additionally, NUTRIOSE^®^ supplementation has been shown to have immunomodulatory effects in mice, rats, piglets, and humans [20,21,22,23,24], and to have effects on genes involved in membrane integrity in rats [23]. 

Colon-on-a-plate^®^ is a high throughput biorelevant ex vivo simulation of the physiology and microbiology of the colon. It has been optimized to perform short-term colonic simulations (up to 48 h) using small volumes, while still allowing an extensive set of readouts, including relative and absolute changes in microbial community composition down to the (sub)species level, impact on microbiome-host cell interactions using secondary human cell assays, and alterations in metabolite production. Where the previous generation of short-term in vitro models struggled with the enrichment of aerotolerant bacteria [25], the Colon-on-a-plate^®^ model has been adapted to ensure anaerobiosis in the reactors. The small assay volumes facilitate the testing of a high number of individuals and/or test conditions, thus enabling to account for interpersonal differences in terms of treatment responses. The latter allows a more accurate prediction of the outcomes of intervention studies in a broad human population, which is considered essential to build product claims.

The primary aim of this study was to assess the indirect effects of NUTRIOSE^®^ on both the host immune response, as measured by chemokine/cytokine release in response to LPS stimulation, and the gut barrier integrity of the host, as measured by transepithelial electrical resistance (TEER), using colonic suspensions obtained with the Colon-on-a-plate^®^ technology platform in an in vitro co-culture model. Secondary aims included the evaluation of fermentation-derived metabolites produced by the gut microbiota during the colonic simulations and to evaluate changes to gut microbial community composition. To assess interindividual variability, fecal samples from eight healthy donors were tested. 

## 2. Materials and Methods

### 2.1. NUTRIOSE^®^ Pre-Digestion

NUTRIOSE^®^ FMHF, a soluble fiber produced from wheat starch, contains 97% of fibers and a small fraction of digestible compounds that are converted to small molecules and absorbed by the small intestine in vivo. Therefore, NUTRIOSE^®^ FM HF was pre-digested using a simulation of the upper gastrointestinal passage (oral, gastric, and small intestine) [26]. For this, a NUTRIOSE^®^ stock solution was prepared in water at 50 g/L. After simulation of small intestinal conditions, the obtained solution was placed inside a dialysis membrane (0.5 kDa pore size), sealed, and dialyzed (dialysis solution: 3.75 g/L NaHCO_3_, pH 7.0) for 24 h at a low temperature (to prevent microbial growth). Dialysis enabled the removal of monosaccharides and disaccharides from the intestinal solution. The blank medium (water) was pre-digested and dialyzed in the same manner. 

### 2.2. Colon-on-a-plate™

The Colon-on-a-plate^®^ system is a miniaturized version of the short-term batch fermentation model, which has proven its ability not only to provide detailed mechanistic insights into the interplay of test products and the human gut microbiota but also to identify their direct and/or indirect effects on host response [27]. With the Colon-on-a-plate^®^ system, multiple test conditions can be simultaneously assessed, either by including a high number of treatments, individuals (to account for interpersonal differences), or combinations thereof. This system utilizes deep well plates, allowing for a ten-fold lower volume per test condition compared with traditional short-term batch fermentations. Its plate format enables the guarantee of identical physical conditions across the experiment, therefore highly benefiting reproducibility.

At the start of the experiment, wells of the Colon-on-a-plate^®^ (24-well plates with 10.4 mL volume-capacity; Thomson, Oceanside, Canada) were filled with 6.3 mL of nutritional medium-fecal inoculum blend (nutritional medium PD001; ProDigest, Gent, Belgium). Next, a single dose (0.7 mL) of pre-digested NUTRIOSE^®^ or blank medium (water, dialyzed according to the procedure described above) was added to each well, bringing the total volume inside the wells to 7 mL, and the theoretical NUTRIOSE^®^ concentration, i.e., not accounting for product losses during dialysis, to 5 g/L (corresponding with 3 g daily administration in a healthy adult person). During each step, anaerobiosis was guaranteed by working in an anaerobic chamber, where oxygen levels were carefully monitored. This study utilized fecal material from 8 healthy individual donors (age range 20–40 years; donors did not use antibiotics during the 3 months preceding stool collection; four participants were male and four were female). Plates were incubated in an anaerobic atmosphere at 37 °C for 48 h. Each condition was tested in triplicate to account for technical variation (8 donors, 1 treatment, 1 blank; each in triplicate). Samples were collected 48 h after the start of the experiment and assessed for pH; community composition; levels of SCFAs (acetate, propionate, and butyrate), lactate, branched SCFAs, and ammonium; and for use in the co-culture experiments to study the effects on TEER and cytokine production.

### 2.3. Caco-2/THP1-Blue™ Co-Culture Model

Co-culture experiments were performed in technical triplicates using Caco-2 (HTB-37, American Type Culture Collection) and THP1-Blue™ cells (InvivoGen, San Diego, CA, USA), as described previously [28]. Briefly, a semi-permeable insert (pore size, 0.4 µM) containing a Caco-2 cell monolayer (TEER > 300 Ω cm^2^ [Epithelial Volt-Ohm meter, Millipore, Burlington, MA, USA]) was placed into a well of phorbol 12-myristate 13-acetic acid (PMA)-differentiated THP1-Blue™ cells (100 ng/mL, 48 h). The apical compartment (Caco-2 cells) was filled with sterile-filtered (0.22 µM) colonic suspensions (diluted 1:5 *v*/*v* in Caco-2 complete medium) or complete Caco-2 medium. The basolateral compartment (THP1-Blue™ cells) was filled with a complete Caco-2 medium. Co-cultures were incubated with humidity at 37 °C, 5% CO_2_ for 24 h, at which time the TEER of each well was measured. The percent of the baseline value (0 h) for each well was calculated after subtracting the empty insert value, using the following formula:

(24 h Ω cm^2^/0 h Ω cm^2^) × 100 (1)

Following the 24 h incubation, the basolateral media was discarded and replaced with Caco-2 complete medium with 500 ng/mL ultrapure LPS (*Escherichia coli* K12, InvivoGen). Basolateral supernatants were collected after 6 h. Human IL-6, IL-10, IL-1β, TNF-α, CXCL10, MCP1, and IL-8 were measured according to the manufacturer’s instructions using Luminex^®^ multiplex (Thermo Fisher Scientific, Waltham, MA, USA).

### 2.4. Microbial Metabolic Activity Analysis

A Senseline F410 pH meter (ProSense, Oosterhout, The Netherlands) was used to measure pH. Acetate, propionate, butyrate, and branched SCFAs (isobutyrate, isovalerate, and isocaproate) were assessed using methods described by De Weirdt et al. [29]. A commercially available enzymatic assay kit (R-Biopharm, Darmstadt, Germany) was used, according to the manufacturer’s instructions, to measure lactate. Ammonium levels were assessed according to the method described by Van de Wiele et al. [30].

### 2.5. Microbial Community Analysis

DNA was extracted as described by Boon et al. [31], implementing modifications as reported by Duysburgh et al. [32]. DNA libraries were prepared using the Nextera XT DNA Library Preparation Kit (Illumina, San Diego, CA, USA) and IDT Unique Dual Indexes with a total DNA input of 1 ng. Genomic DNA was fragmented using a proportional amount of Illumina Nextera XT fragmentation enzyme. Unique dual indexes were added to each sample, followed by 12 cycles of PCR to construct libraries. DNA libraries were purified using AMpure magnetic beads (Beckman Coulter, Brea, CA, USA) and eluted in QIAGEN EB buffer (Germantown, MD, USA). DNA libraries were quantified using a Qubit™ 4 fluorometer and the Qubit™ dsDNA HS Assay Kit (Thermo Fisher, Waltham, MA, USA). Unassembled sequencing reads were directly analyzed for multi-kingdom microbiome analysis and quantification of relative abundances as previously described [33,34,35,36]. Briefly, curated genome databases combined with a high-performance data-mining algorithm were used to disambiguate hundreds of millions of metagenomic sequence reads into the specific microorganisms that contain the particular sequences in their DNA. Total cell counts in the various samples were determined using a BD Accuri C6 Plus Flow Cytometer (BD Biosciences, Franklin Lakes, NJ, USA) using the high flow rate setting with a threshold of 700 on the SYTO channel. To account for differences in bacterial biomass across the samples, relative abundances of each population in a sample were multiplied with the total cell count obtained by flow cytometry for a given sample, to convert proportional values obtained using shotgun sequencing to absolute quantities.

### 2.6. Statistical Methods

Metabolite production was compared between colonic suspensions from NUTRIOSE^®^-supplemented and blank wells, using paired two-tailed student’s *t*-tests. To do so, averages of technical replicates were calculated for each condition and per-donor measurements were used as replicate values for the statistical tests. As eight donors were included, eight replicates were considered, thus providing good statistical power. By applying this methodology, an effect was considered significant only if it was observed across multiple individuals. A *p*-value of <0.05 was considered statistically significant. 

To evaluate differences in TEER and immune markers, per donor, data for NUTRIOSE^®^-supplemented colonic suspensions were compared to their non-supplemented blank controls using a two-way ANOVA with Sidak’s multiple comparisons test. Separately, the average of all donor treatment samples was compared to the average of the blank controls, using an unpaired, two-tailed student’s *t*-test. 

For analysis of community composition, differential abundance analysis using treeclimbR [37] was performed to identify the taxa most likely to explain differences between NUTRIOSE^®^-supplemented and blank conditions. The resulting volcano plots show statistical significance (i.e., *p*-value) on the y-axis versus the magnitude of change (i.e., fold change) on the x-axis. Thus, the scatterplot classifies taxa into the following four categories based on abundance in compared treatments: (a) not significant and not biologically relevant, (b) biologically relevant, but not statistically significant, (c) statistically significant, but not biologically relevant, and d) biologically and statistically significant. For this analysis, a *p*-value of <0.05 was considered statistically significant, and a >4-fold change was considered biologically significant. 

Statistical analyses to evaluate differences in metabolite production, as well as TEER and immune parameters were performed using GraphPad Prism version 9.3.1 for Windows (GraphPad Software, San Diego, CA, USA). Statistical analysis on community composition was performed in R.

## 3. Results

### 3.1. Analysis of Host-Microbe Interactions

#### 3.1.1. Transepithelial Electrical Resistance

**Figure 1:**
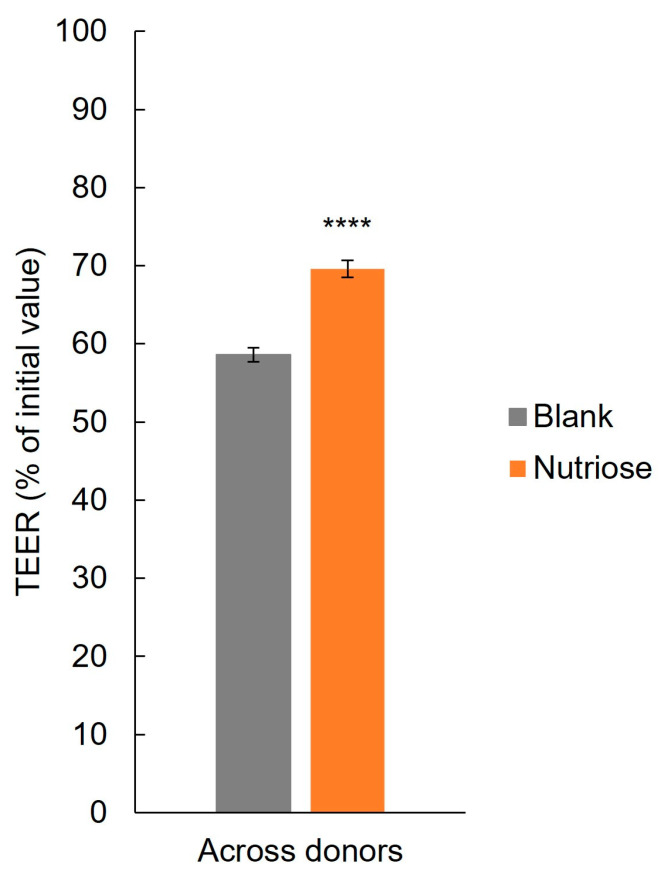
Barrier integrity of Caco-2 cells after exposure to colonic suspensions. **** *p* < 0.0001 for differences between the NUTRIOSE^®^-supplemented and blank samples. Data are plotted as mean (all 8 donors) ± standard error of the mean; TEER = transepithelial electric resistance.

Across all donors, TEER was significantly increased with NUTRIOSE^®^ supplemented colonic suspensions versus blank (*p* < 0.0001) (Figure 1), demonstrating protection of the intestinal epithelial barrier from inflammation-induced disruption. For individual donors, TEER was increased with NUTRIOSE^®^ supplemented colonic suspensions versus blank for each donor and reached significance in donors B, C, D, and F (Supplemental Figure S1).

#### 3.1.2. Immune Markers

**Figure 2:**
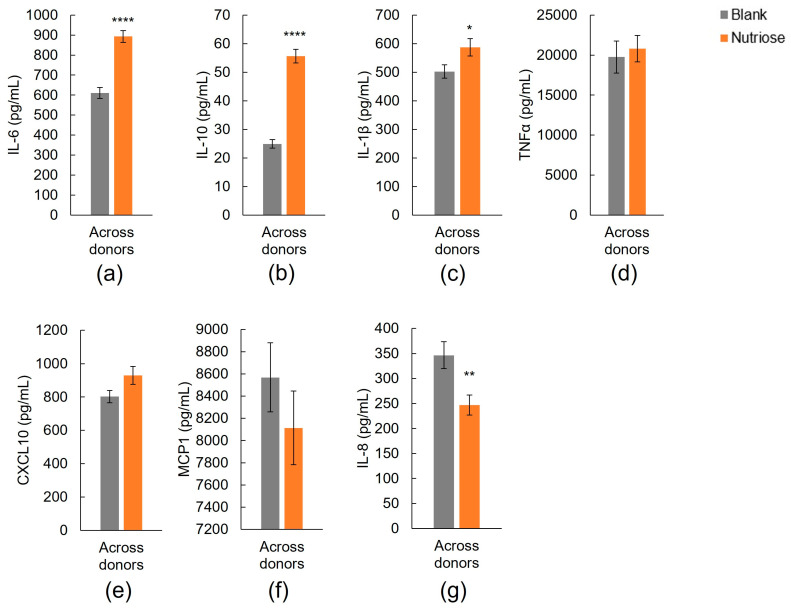
Effect of colonic suspensions on release of (**a**) IL-6, (**b**) IL-10, (**c**) IL-1β, (**d**) TNF-α, (**e**) CXCL10, (**f**) MCP1, and (**g**) IL-8 by PMA-treated THP1-blue™ cells after LPS stimulation in the Caco-2/THP1-blue™ co-culture model. * *p* < 0.05, ** *p* < 0.01, **** *p* < 0.0001 for differences between the NUTRIOSE^®^-supplemented and blank samples. Data are plotted as mean (all 8 donors) ± standard error of the mean. LPS = lipopolysaccharide.

Across all donors, there was a significant increase in the secretion of IL-6 (both pro-and anti-inflammatory cytokine) and of the anti-inflammatory cytokine IL-10 with NUTRIOSE^®^-supplemented colonic suspensions versus blank suspensions (*p* < 0.0001 for both) (Figure 2a,b, respectively). For individual donors, a significant increase in IL-6 or IL-10 was observed for five of eight or all donors, respectively (Supplemental Figure S2a,b, respectively).

A significant increase in the pro-inflammatory cytokine IL-1β was observed with NUTRIOSE^®^-supplemented colonic suspensions versus blank suspensions with all donors combined (*p* < 0.05) (Figure 2c) and for a single donor (Donor H), though non-significant increases were observed in five other donors (Supplemental Figure S2c). Across all donors, levels of the pro-inflammatory cytokines TNF-α, CXCL10, and MCP1 were not increased with NUTRIOSE^®^-supplemented colonic suspensions versus blank suspensions (Figure 2d,e,f, respectively) and levels of IL-8 were significantly decreased (*p* < 0.01) (Figure 2g). None of the individual donors demonstrated an increase in TNF-α (Supplemental Figure S2d), one donor (Donor B) had a significant increase in CXCL10 (Supplemental Figure S2e), none had an increase in MCP1 (Supplemental Figure S2f), and one (Donor h) had a significant decrease in IL-8 and non-significant decreases were observed for three other donors (Supplemental Figure S2g) with NUTRIOSE^®^-supplemented colonic suspensions versus blank suspensions.

### 3.2. Microbial Community Analysis

#### 3.2.1. Fermentation Activity and Changes in Metabolite Production

**Figure 3:**
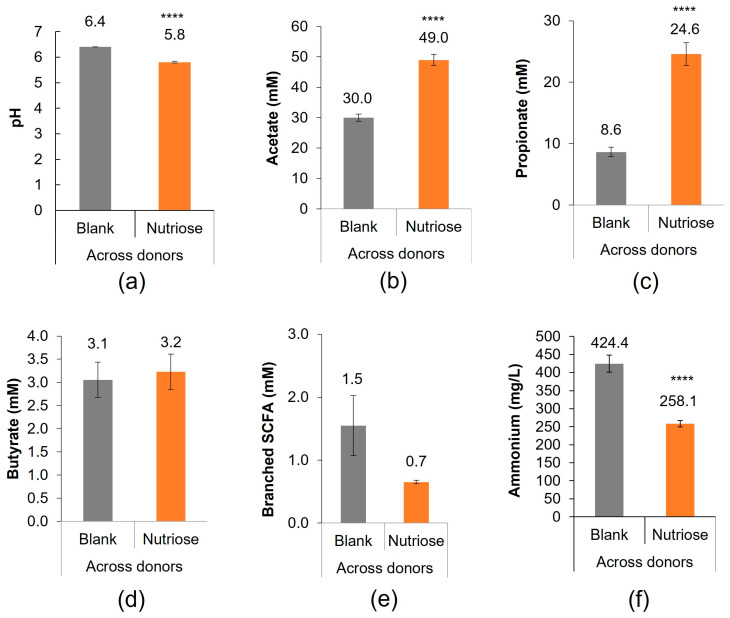
Overall microbial community activity (acidification) and microbial metabolic activity shown as (**a**) pH, (**b**) acetate, (**c**) propionate, (**d**) butyrate, (**e**) branched SCFA, and (**f**) ammonium at 48 h. Measurements were collected in triplicate. **** *p* < 0.0001 for differences between the NUTRIOSE^®^-supplemented and blank samples. Data are plotted as mean (all 8 donors) ± standard error of the mean. SCFA = short-chain fatty acid.

Decreases in pH indicate an increase in bacterial fermentation. pH measurements at 48 h indicated greater fermentation activity with NUTRIOSE^®^ supplementation versus blank (*p* < 0.0001) (Figure 3a). pH results for individual donors are shown in Supplemental Figure S3a.

SCFA levels (acetate, propionate, and butyrate), which represent carbohydrate metabolism in the colon, were measured at 48 h. Across all donors, SCFA levels with NUTRIOSE^®^ supplementation versus blank were increased for acetate (*p* < 0.0001; Figure 3b) and propionate (*p* < 0.0001; Figure 3c), and similar for butyrate (*p* = 0.487; Figure 3d). SCFA results for individual donors are shown in Supplemental Figure S3b–d.

Markers of protein metabolism were also measured. Across all donors, levels of branched SCFAs were numerically decreased but did not reach statistical significance (*p* = 0.09; Figure 3e) and levels of ammonium were lower (*p* < 0.0001; Figure 3f) with NUTRIOSE^®^ supplementation versus blank. Changes in branched SCFAs and ammonium for individual donors are shown in Supplemental Figure S3e,f.

#### 3.2.2. Changes in Microbial Community Composition

**Figure 4:**
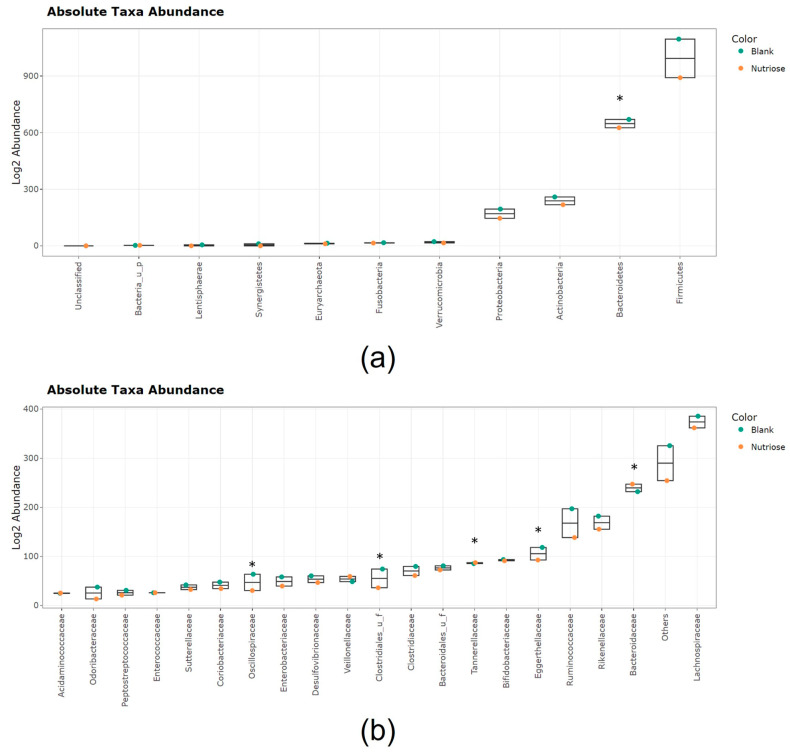
Jitter plots showing average abundances (log2 abundances) at 48 h shown as (**a**) phylum and (**b**) family (20 most abundant families; the sum of abundances of the remaining families is categorized as ‘others’). Data for average values were derived using data from all 8 donors. Orange circles represent NUTRIOSE^®^-supplemented colonic microbiota and the turquoise circles represent blank colonic microbiota. UF, unidentified family. Asterisks indicate phyla/families that were differentially abundant in blank and NUTRIOSE^®^ condition, for which statistical significance was reached based on treeclimbR.

Jitter plots showing the effects of NUTRIOSE^®^ supplementation on the microbial abundances across all donors at different taxonomic levels are shown in Figure 4. The most abundant phylum with both NUTRIOSE^®^ supplementation and blank was Firmicutes, followed by Bacteroidetes, Actinobacteria, and Proteobacteria (Figure 4a). 

Within the Firmicutes phylum, the most abundant families were *Lachnospiraceae* (comprising acetate-, propionate-, and succinate-producers) and *Ruminococcaceae* (comprising acetate-, lactate-, succinate-, and butyrate-producers); levels of these families were higher with blank than with NUTRIOSE^®^ supplementation (Figure 4b). The Bacteroidetes phylum included *Bacteroidaceae* (comprising acetate-, propionate-, and succinate-producers), *Rikenellaceae* (comprising succinate-producers), and *Tannerellaceae* (comprising acetate-, succinate-, and in some cases propionate-producers); *Bacteroidaceae* and *Tannerellaceae* were significantly increased with NUTRIOSE^®^ supplementation versus blank. Actinobacteria were mostly represented by the families *Eggerthellaceae* (comprising acetate-producers) and *Bifidobacteriaceae* (comprising acetate- and lactate-producers); for these families, abundances were higher with blank than with NUTRIOSE^®^ supplementation. The Proteobacteria phylum was mostly represented by *Enterobacteriaceae* and *Desulfovibrionaceae*; abundances were higher with the blank than with NUTRIOSE^®^ supplementation.

**Figure 5:**
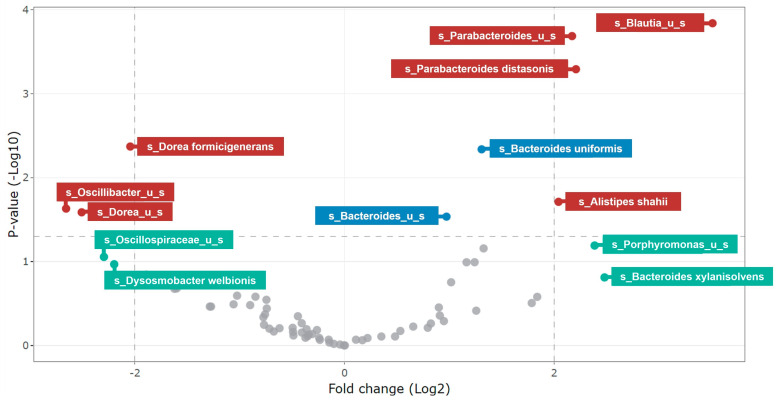
Differential abundance analysis at 48 h using treeblimbR. A *p*-value of < 0.05 is considered statistically significant and a >4-fold change is considered biologically significant. Maroon circles represent biologically and statistically significant changes, turquoise circles represent biologically significant but not statistically significant changes; gray circles represent non-significant changes; and blue circles represent statistically significant but not biologically significant changes. US = unidentified species.

Differential abundance analysis revealed statistically significant (*p* < 0.05) and biologically significant (>4-fold) enrichment with NUTRIOSE^®^ supplementation versus blank for *Parabacteroides distasonis* (acetate- and succinate-producer), *Alistipes shahii* (succinate-producer), an unidentified *Parabacteroides* species (acetate- and succinate-producing genus), and an unidentified *Blautia* species (acetate-, lactate-, succinate-, and/or butyrate-producing genus) (Figure 5). The enrichment of each of these bacteria was considered relevant from a biological point of view and was observed across a multitude of donors (the threshold for statistical significance for across-donor comparisons was reached). Biologically but not statistically significant enrichment was observed for a member of the genus *Porphyromonas* (acetate-, propionate-, butyrate-, and branched SCFA producers) and *Bacteroides xylanisolvens* (acetate-, succinate-, and propionate-producer) with NUTRIOSE^®^ supplementation versus blank, implying that due to interindividual differences, these enrichments were not observed in all donors, but the magnitude of the enrichment (effect size) in at least one donor was considered relevant from a biological point of view (on average, the fold change was >4). There were also several enrichments that were statistically significant but not biologically significant for NUTRIOSE^®^ supplementation versus blank, meaning that those enrichments were observed across multiple donors, but the sizes of the effect were small. Hence, whether such enrichments would be translated into alterations in metabolite levels is questionable. These enrichments included members of the genus *Bacteroides* (acetate-, succinate-, and propionate-producers), particularly *Bacteroides uniformis* and an unidentified *Bacteroides* species. In contrast, several bacteria were more abundant in blank than NUTRIOSE^®^ condition, involving members of bacterial genera *Dorea*, *Oscillibacter*, and *Dysosmobacter*, meaning that these bacteria were not enriched upon treatment with NUTRIOSE^®^.

#### 3.2.3. Metabolite-Metagenomics Correlations

**Figure 6:**
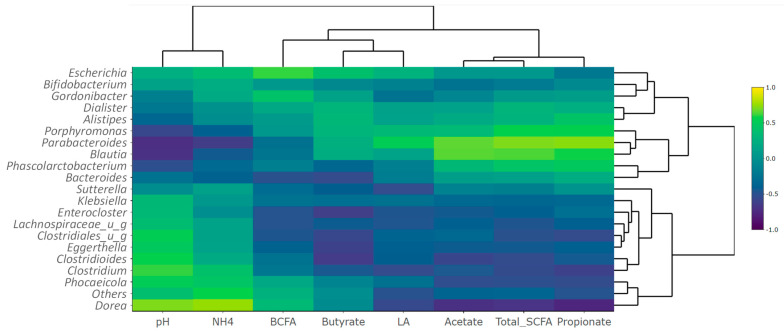
Metabolite-taxon correlation plot at the genus level (20 most abundant genera are shown). Hierarchical clustering of columns (metabolites) and rows (taxa) of the metabolite-taxon heatmaps is based on Euclidean distances (high-dimensional). The heatmaps were made into dendrograms using complete-linkage clustering. UG = unidentified genus.

Correlations between metabolite production and taxa abundances were assessed using heatmaps to generate metabolite-taxon correlation plots. At the genus level, there was a strong correlation between the production of acetate and the enrichment of *Blautia* and *Parabacteroides* (Figure 6). Additionally, there was a strong correlation between propionate production and the enrichment of *Parabacteriodes*, *Bacteroides*, *Porphyromonas*, *Blautia*, *Phascolarctobacterium*, and *Alistipes* species. Finally, a positive correlation was found for the production of BCFA and the growth of *Escherichia*, and for the production of ammonium and the growth of *Dorea*.

Some of these correlations reveal direct effects of microbial enrichments on metabolite levels in the reactors, including the acetogenic effect of *Blautia* and *Parabacteroides*, whereas other positive correlations are indirect, including the propionogenic effect of *Parabacteroides*, whereby *Parabacteroides*, through the production of succinate, promotes the propionate-synthesis via the succinate pathway. Others, like the correlation between *Dorea* and the production of ammonium, resulted from a cascade of interactions with intestinal bacteria, as the bacterial genus *Dorea* does not encode the production of ammonium in its genome (ammonium is produced by urease-producing bacteria).

## 4. Discussion

Using pre-clinical models, colonic fermentation of NUTRIOSE^®^ demonstrated protective effects on host functions by reducing inflammation-induced intestinal barrier damage and increasing the anti-inflammatory response to LPS with all eight donors. Mostly minor effects were seen on the pro-inflammatory response, with the exception of IL-8 which was decreased with four of the eight donors. The host-microbe interactions were facilitated by the fermentation of NUTRIOSE^®^ by the donor gut microbiota, which resulted in increased acetate and propionate production and decreased ammonium production. The acetate and propionate increases are likely explained by the enrichment of SCFA-producing bacteria observed with NUTRIOSE^®^ supplementation. 

The intestinal epithelial barrier is formed by intercellular tight junctions and functions to control the trafficking of molecules from the intestinal lumen to the lamina propria [38,39]. Dysregulation of this barrier can result in ‘leaky gut’, where molecules, including bacterial products such as LPS, are inappropriately trafficked from the intestinal lumen to the lamina propria, resulting in inflammation [39]. This dysregulation is associated with multiple disorders, including obesity and metabolic syndrome [40,41], and autoimmune disorders [42,43]. A significant increase in the expression of the genes encoding the tight junction proteins occludin and ZO-1 was observed in the colons of rats who received NUTRIOSE^®^ supplementation versus those who did not [23,24,44], indicating that NUTRIOSE^®^ may improve the intestinal epithelial barrier. Those findings are supported by the present study, which found that exposure to colonic fermentations following NUTRIOSE^®^ supplementation resulted in higher TEER values (i.e., a stronger intestinal epithelial barrier) versus blank colonic fermentations in the co-culture model. Thus, the fermentation products of NUTRIOSE^®^ provided protection against inflammation-mediated widening of tight junctions. This not only shows the protective impact of NUTRIOSE^®^ at the level of the intestinal epithelial barrier but also the capacity of the Colon-on-a-plate^®^ technology coupled with the co-culture model to predict indirect treatment effects on the host that were obtained during an animal trial. Using similar in vitro models, exposure of Caco-2/THP-1 cocultures to colonic fermentations from other well-established oligosaccharide prebiotics including arabinogalactan, arabinoxylo-oligosaccharide (AXOS), inulin, and a formulation including fructo-oligosaccharides (FOS), xylo-oligosaccharide (XOS), and galacto-oligosaccharides (GOS), has been reported to significantly increase Caco-2 cell TEER values versus blank colonic fermentations in response to activated THP1 cells [28,45,46]. Similarly, it is reported that direct exposure of Caco-2 cells to prebiotic oligosaccharides GOS or FOS resulted in increased TEER values versus mock exposed Caco-2 cells (GOS: +33.62%, *p* = 0.00037; FOS: +28.68%, *p* = 0.054) [47]. 

NUTRIOSE^®^ also had an immunomodulatory effect on cytokine production. Levels of IL-6 (both pro- and anti-inflammatory properties) and of the anti-inflammatory cytokine IL-10 were increased (5/8 donors and 8/8 donors, respectively) and the levels of the pro-inflammatory chemokine IL-8 were decreased (4/8 donors) with exposure to NUTRIOSE^®^-supplemented versus blank colonic fermentations. The immune-related findings reported in the present study are largely supported by previous studies of NUTRIOSE^®^ supplementation in humans, rats, and mice. Two clinical studies reported increased serum levels of anti-inflammatory cytokines (IL-4 and IL-10) and decreased levels of TNF-α, IFNγ, IL-12, and IL-6 in females with type 2 diabetes who received 8 weeks of daily NUTRIOSE^®^ supplementation (10 g/day) versus baseline levels [21,22]. A decrease (versus control) in the expression of TNF-α or IL-1β in the colons of rats or mice, respectively, has also been reported with NUTRIOSE^®^ supplementation [23]. Together, these findings indicate that NUTRIOSE^®^ has strong anti-inflammatory effects, and further demonstrate the predictivity of the Colon-on-a-plate^®^ and the applied co-culture models for human and animal trials. Immunomodulatory effects on cytokine production have also been reported for other prebiotics using in vitro models. For example, Caco-2/THP1 cocultures exposed to colonic fermentations of arabinogalactan or FOS produced significantly more IL-10 (arabinogalactan only) and significantly less IL-8 following LPS stimulation compared with cocultures exposed to blank colonic fermentations [28]. Cocultures exposed to colonic fermentations of inulin or AXOS produced significantly more IL-10 and IL-6 in response to LPS stimulation versus blank colonic fermentations [45], as did cocultures exposed to colonic fermentations of a formulation including FOS, XOS, and GOS, as well as spore-based probiotics, immunoglobulin A, and amino acids [46]. The similar in vitro findings (increased TEER, increased production of IL-10 and IL-6, and decreased production of IL-8) for NUTRIOSE^®^ and well-studied prebiotics such as FOS, GOS, and inulin, which are well described for their support of the immune system [48,49,50,51,52], indicate that NUTRIOSE^®^ supplementation will also provide immune system support. 

To understand the underlying mechanisms by which NUTRIOSE^®^ fermentation imparts these immunomodulatory effects, the outcomes of NUTRIOSE^®^ supplementation on the microbial community activity and composition were also evaluated. Changes in pH with supplementation indicated that NUTRIOSE^®^ was well fermented by the gut microbiota of all eight donors. The production of acetate and propionate was consistently increased with supplementation across all donors. Levels of butyrate production were not apparently affected by NUTRIOSE^®^ supplementation. A preclinical study of NUTRIOSE^®^ supplementation in rats demonstrated increased production of acetate, propionate, and butyrate with supplementation versus without [16]. The difference in butyrate production between these studies may be explained by the different experimental methods. Both propionate and acetate have beneficial effects on the host. Propionate alters inflammatory gene expression in colonic epithelial cells, downregulating IL-8 and MCP-1 expression and potentially contributing to local containment of immune responses [53]. Additionally, it is well established that propionate and acetate promote gut barrier function and intestinal epithelial cell integrity [54,55,56,57], and that SCFAs induce the expression of tight junction proteins, including occludin [56]. The enrichment of succinate- (an intermediate metabolite in propionate synthesis [58]), acetate-, propionate-, and/or butyrate-producing bacterial species, including *P. distasonis*, *A. shahii*, and members of the Bacteroidetes phylum with NUTRIOSE^®^ supplementation supports the metabolic findings. Indeed, metabolite-taxa correlation analysis demonstrated a link between acetate production and the enrichment of the strictly anaerobic *Blautia* and *Parabacteroides* genera, and between propionate production and the enrichment of the *Parabacteroides*, *Bacteroides*, *Porphyromonas*, *Blautia*, *Phascolarctobacterium*, and *Alistipes* genera. Similar changes in the microbial community composition with NUTRIOSE^®^ supplementation have been observed in clinical studies. For example, NUTRIOSE^®^ supplementation (8 g/day or 10 g/day for 14 days) in healthy volunteers resulted in an increase (day 14 vs. baseline) in *Bacteroides* and propionate-producing bacteria, as well as members of the *Parabacteroides* genus [17,18]. Additionally, a recent study of healthy females who were supplemented with NUTRIOSE^®^ (increasing doses of 5, 10, and 20 g/day) or a control product for 6 weeks found a significant increase in *P. distasonis* (NUTRIOSE^®^ versus control) [19]. The significant enrichment of *P. distasonis* observed across the various donors in the ex vivo simulation thus accurately reflects the result of a clinical trial as detailed as the bacterial species level. The outcome of these clinical trials therefore strongly correlates with the results obtained with the Colon-on-a-plate^®^ technology confirming the effects of NUTRIOSE^®^ supplementation on the microbial community composition, and the validity of the Colon-on-a-plate^®^ as an accurate simulation of the gut microbiota.

To make the connection between microbiota modulation and metabolite production with the production of cytokines, it would have been interesting to study the toll-like receptor signal transduction through different intracellular molecules such as MAP kinases or nuclear factor kappa B (NFkB). As with any other in vitro/ex vivo study, our findings are limited in that they cannot directly translate to a biological response. However, the data presented herein further support the prebiotic properties of NUTRIOSE^®^ for human gut microbes.

## 5. Conclusions

This study demonstrated that the Colon-on-a-plate^®^ technology accurately predicts the outcome of clinical and animal trials with respect to direct prebiotic effects and indirect effects on the host, including inflammatory response and epithelial integrity. Using this ex vivo methodology, the present study confirmed that NUTRIOSE^®^ is well fermented by members of the Bacteroidetes and Firmicutes phyla, inducing the production of bioactive molecules, including acetate and propionate. It is these metabolites that interacted with the colonic epithelial cells, likely explaining the protective effect of supplementation on inflammation-induced disruption of the intestinal epithelial barrier. Supplementation was also associated with immune-modulatory properties and strong anti-inflammatory effects. This means that it was NUTRIOSE^®^’s fermentation products, rather than unfermented NUTRIOSE^®^, that was responsible for the effects on barrier integrity and immune regulation. Given these properties, NUTRIOSE^®^ may be a promising candidate for increasing the defense against pathogens and protecting against inflammation in the gastrointestinal tract.

## Data Availability

Data are unavailable due to privacy and ethical restrictions.

## Supplementary Materials

The following supporting information can be downloaded at: https://www.mdpi.com/article/10.3390/nu15194229/s1, Figure S1: Barrier integrity of Caco-2 cells after exposure to colonic suspensions; Figure S2: Effect of colonic suspensions on measures of immune response; Figure S3: Overall microbial community activity (acidification) and microbial metabolic activity at 48 h.
