# Supplementary material for: Ex Vivo Colonic Fermentation of NUTRIOSE® Exerts Immuno-Modulatory Properties and Strong Anti-Inflammatory Effects
Source: Nutrients. 2023 Sep 30;15(19):4229. doi: 10.3390/nu15194229 (PMC10574048; doi:10.3390/nu15194229)
Supplement: Supplementary file 1 [file nutrients-15-04229-s001.zip › nutrients-2550171-supplementary.pdf]

Supplemental Material

Ex Vivo Colonic Fermentation of NUTRIOSE® Exerts Immuno-Modulatory Properties and Strong Anti-Inflammatory Effects

Caroline Perreau [1], Clementine Thabuis [1], Lynn Verstrepen [2], Jonas Ghyselinck [2] and Massimo Marzorati [2,3,*]

Figure S1. Barrier integrity of Caco-2 cells after exposure to colonic suspensions. *$p<0.05$ for differences between the NUTRIOSE®-supplemented and blank samples for co-cultures treated with CM. Data are plotted as mean (each individual donor) ± standard error of the mean. CM = Caco-2 media; TEER = transepithelial electric resistance.

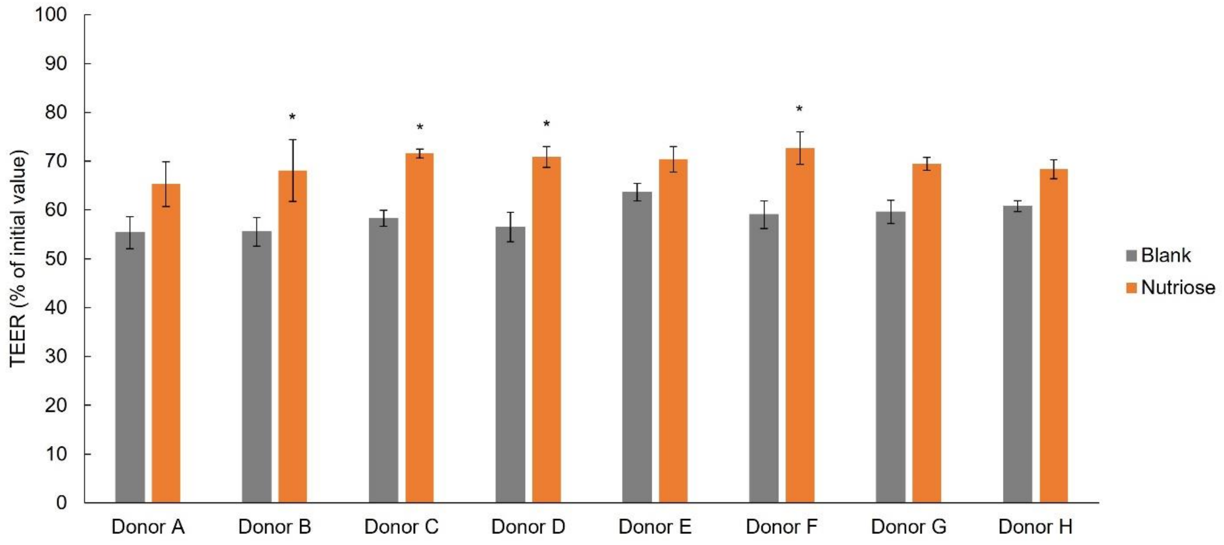

Figure S2. Effect of colonic suspensions on measures of immune response. (a) IL-6, (b) IL-10, (c) IL-1β, (d) TNFα, (e) CXCL10, (f) MCP1, and (g) IL-8 release from PMA-treated THP1-blue™ cells after LPS stimulation in the Caco-2/THP1-blue™ co-culture model for individual donors. *p<0.05, **p<0.01, ***p<0.001, ****p<0.0001 for differences between the NUTRIOSE®-supplemented and blank samples. Data are plotted as mean (all 8 donors) ± standard error of the mean. LPS = lipopolysaccharide.

(a)

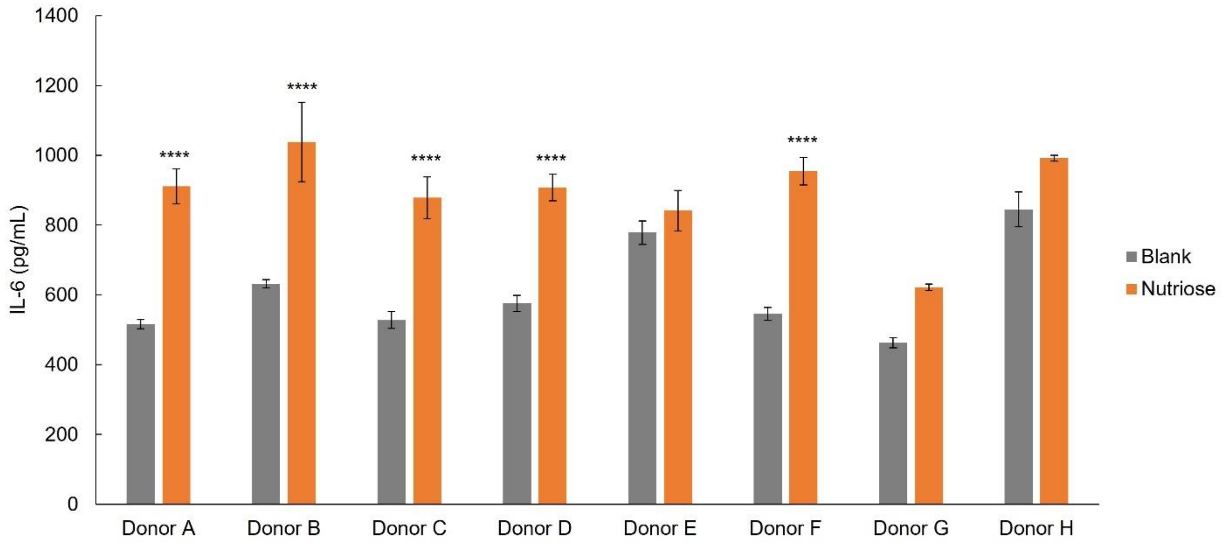

(b)

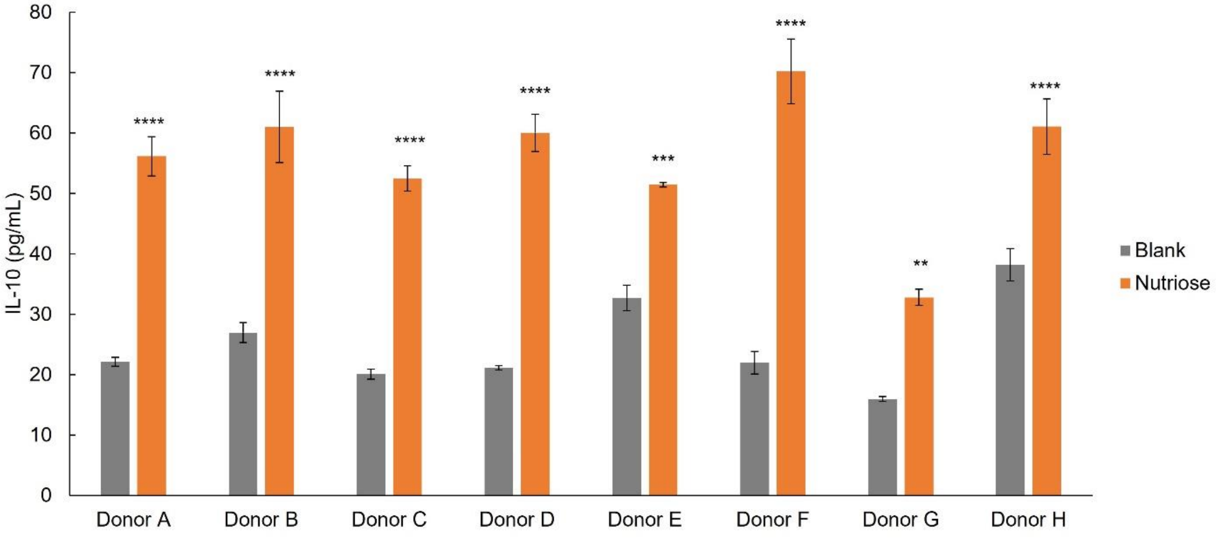

80
70
60
50
40
30
20
10
0
IL-10 (pg/mL)
****
****
****
****
***
****
**
****
Donor A
Donor B
Donor C
Donor D
Donor E
Donor F
Donor G
Donor H
Blank
Nutriose

(c)

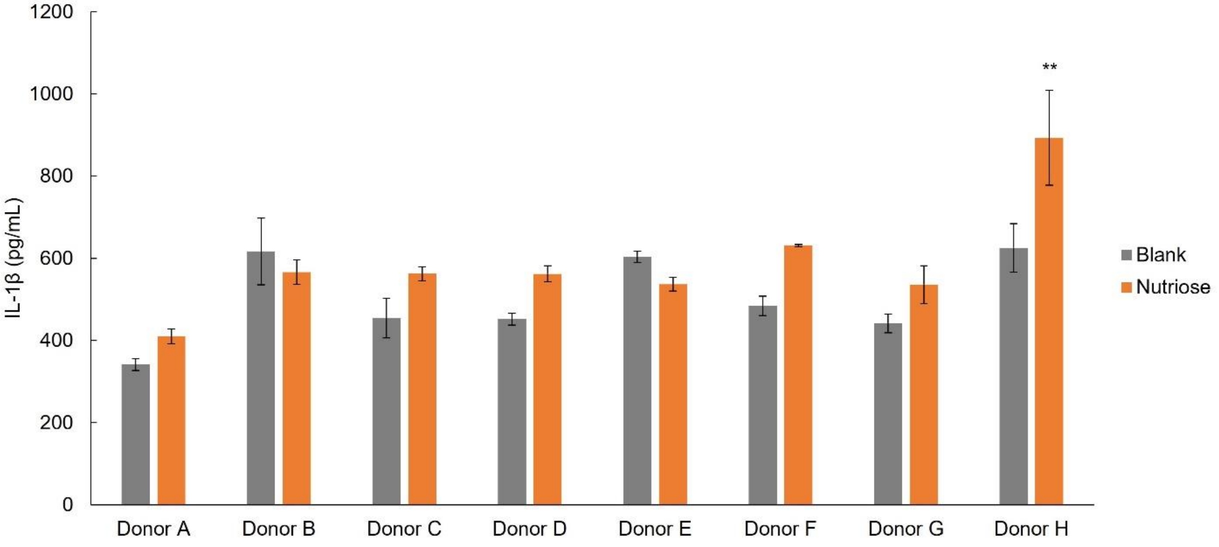

1200
1000
800
600
400
200
0
IL-1β (pg/mL)
**
Donor A
Donor B
Donor C
Donor D
Donor E
Donor F
Donor G
Donor H
Blank
Nutriose

(d)

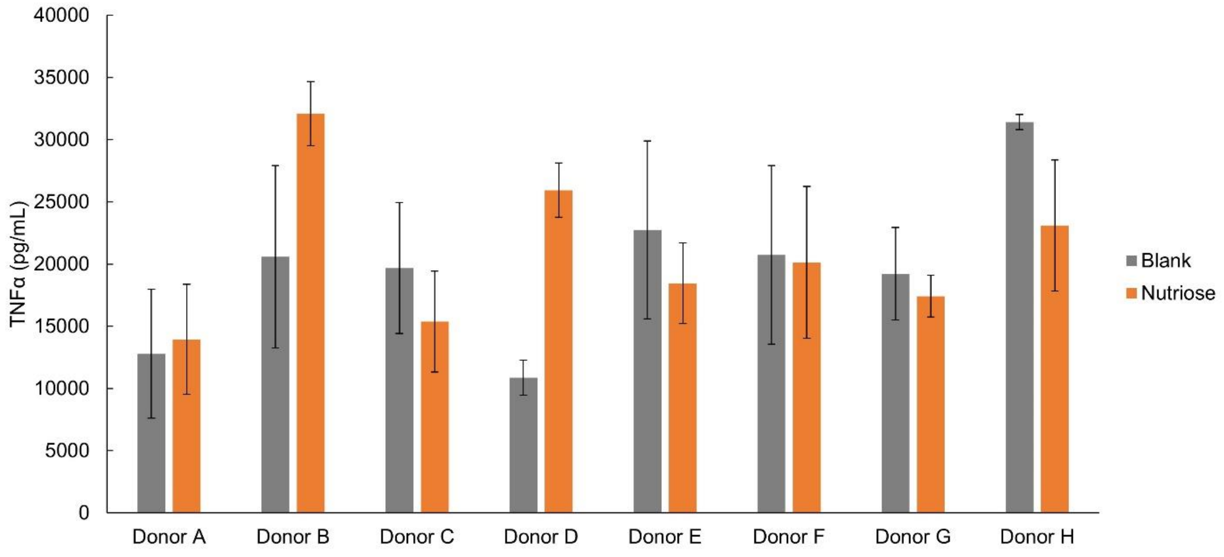

40000
35000
30000
25000
20000
15000
10000
5000
0
TNFα (pg/mL)
Donor A
Donor B
Donor C
Donor D
Donor E
Donor F
Donor G
Donor H
Blank
Nutriose

(e)

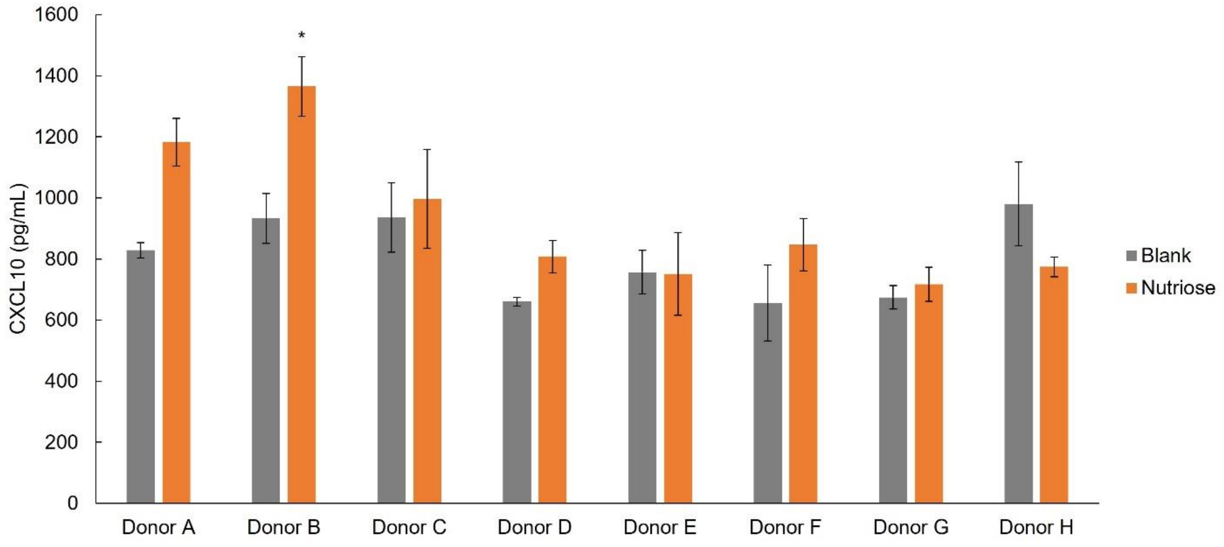

1600
1400
1200
1000
800
600
400
200
0
CXCL10 (pg/mL)
*
Donor A
Donor B
Donor C
Donor D
Donor E
Donor F
Donor G
Donor H
Blank
Nutriose

(f)

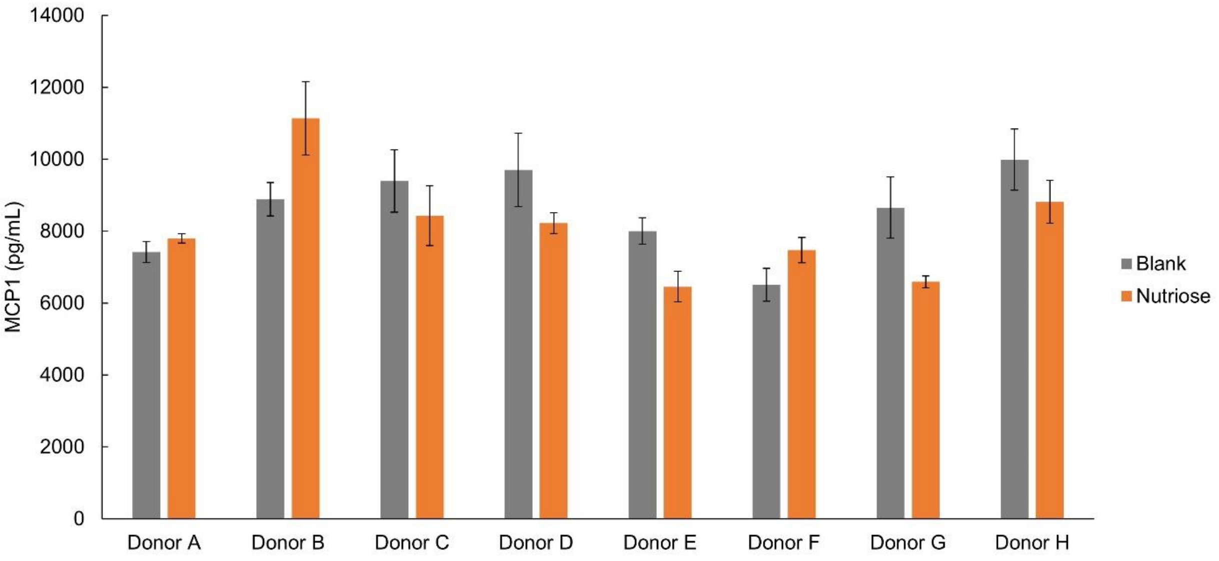

14000
12000
10000
8000
6000
4000
2000
0
MCP1 (pg/mL)
Donor A
Donor B
Donor C
Donor D
Donor E
Donor F
Donor G
Donor H
Blank
Nutriose

(g)

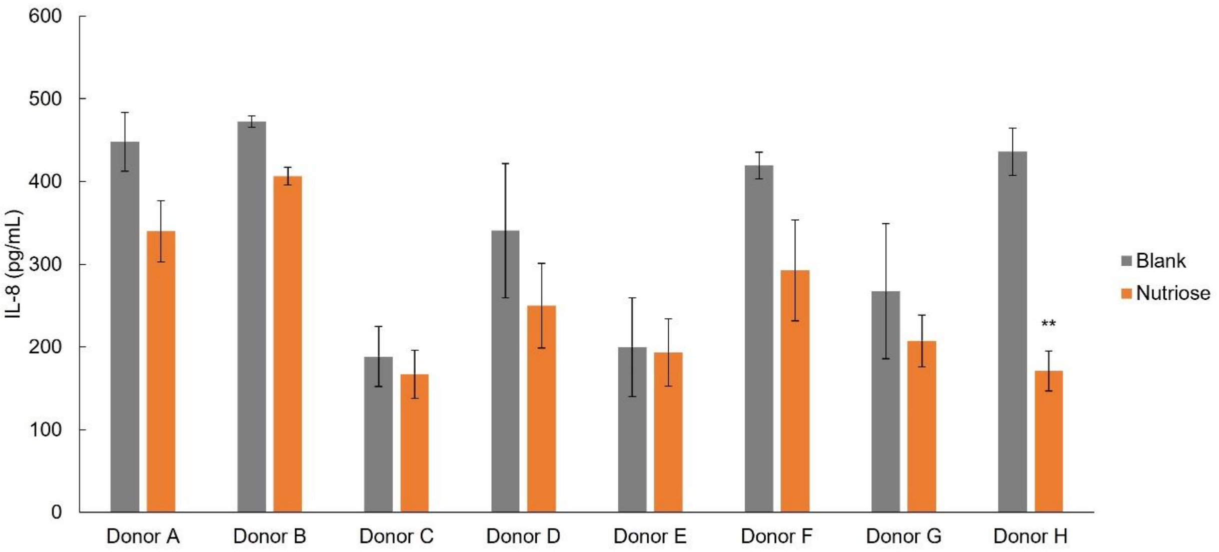

600
500
400
300
200
100
0
IL-8 (pg/mL)
**
Donor A
Donor B
Donor C
Donor D
Donor E
Donor F
Donor G
Donor H
Blank
Nutriose

Figure S3. Overall microbial community activity (acidification) and microbial metabolic activity at 48h shown as (a) pH, (b) acetate, (c) propionate, (d) butyrate, (e) branched SCFA, and (f) ammonium for individual donors. Measurements were collected in triplicate. Error bars represent standard deviation. SCFA = short-chain fatty acid.

(a)

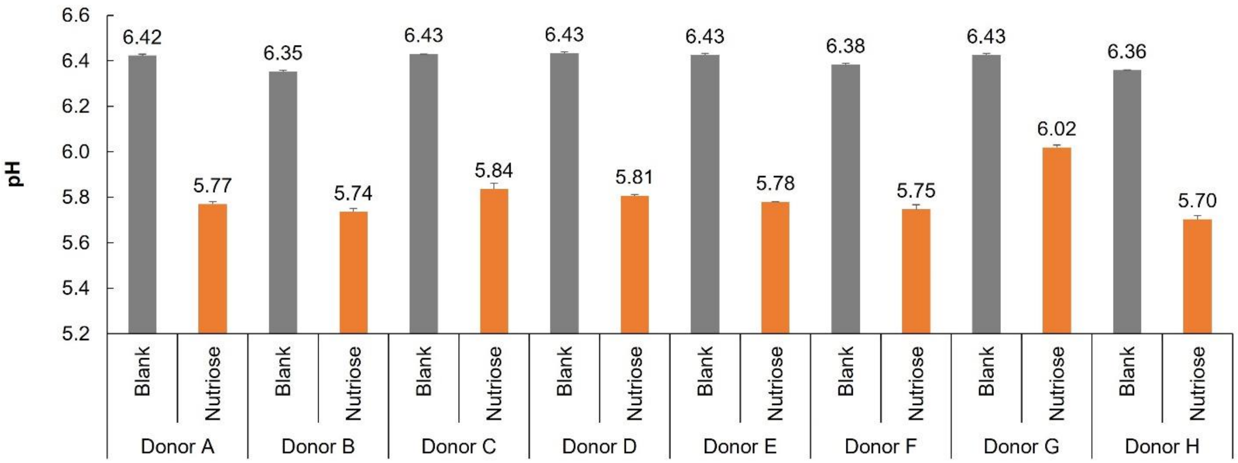

(b)

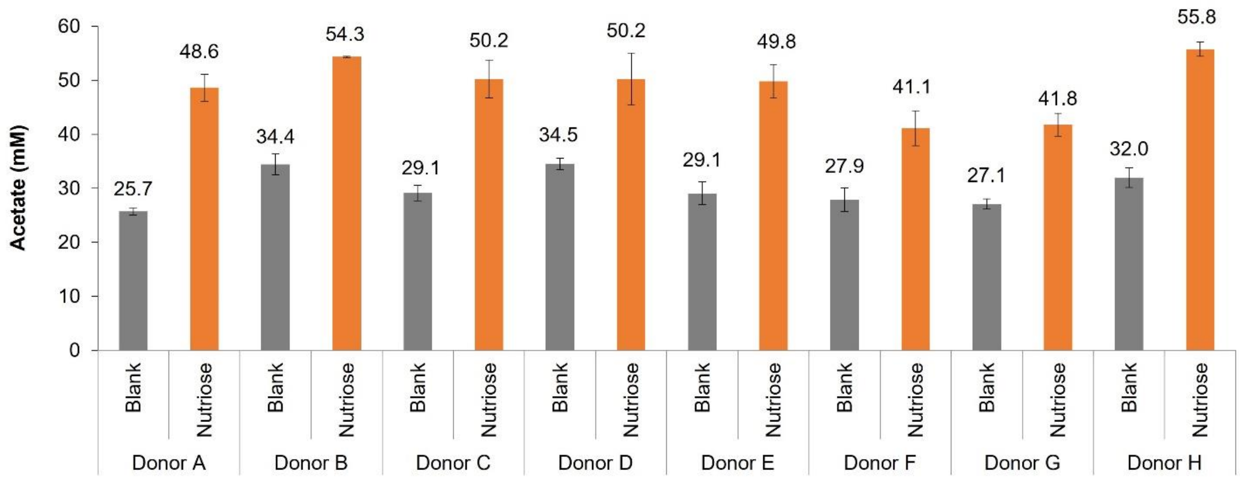

Acetate (mM)
60
50
40
30
20
10
0
25.7
48.6
34.4
54.3
29.1
50.2
34.5
50.2
29.1
49.8
27.9
41.1
27.1
41.8
32.0
55.8
Blank
Nutriose
Donor A
Donor B
Donor C
Donor D
Donor E
Donor F
Donor G
Donor H

(c)

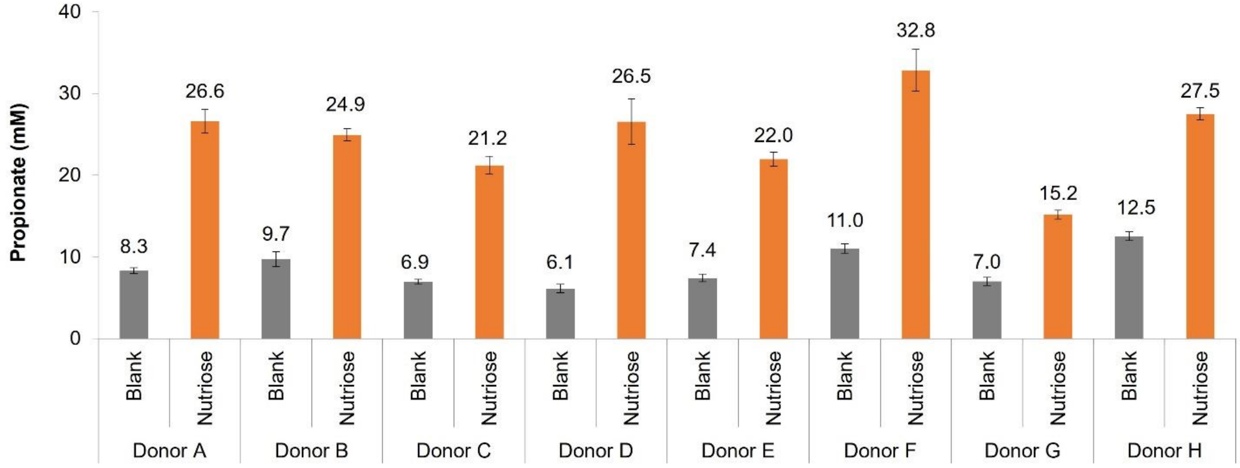

Propionate (mM)
40
30
20
10
0
8.3
26.6
9.7
24.9
6.9
21.2
6.1
26.5
7.4
22.0
11.0
32.8
7.0
15.2
12.5
27.5
Blank
Nutriose
Donor A
Donor B
Donor C
Donor D
Donor E
Donor F
Donor G
Donor H

(d)

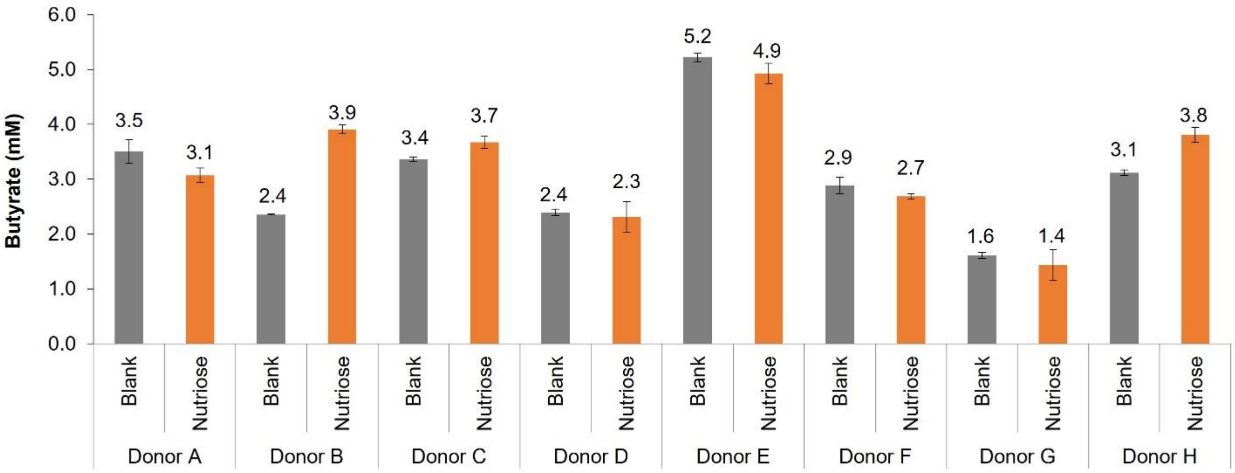
Butyrate (mM)
6.0
5.0
4.0
3.0
2.0
1.0
0.0
3.5
3.1
2.4
3.9
3.4
3.7
2.4
2.3
5.2
4.9
2.9
2.7
1.6
1.4
3.1
3.8
Blank
Nutriose
Donor A
Donor B
Donor C
Donor D
Donor E
Donor F
Donor G
Donor H

(e)

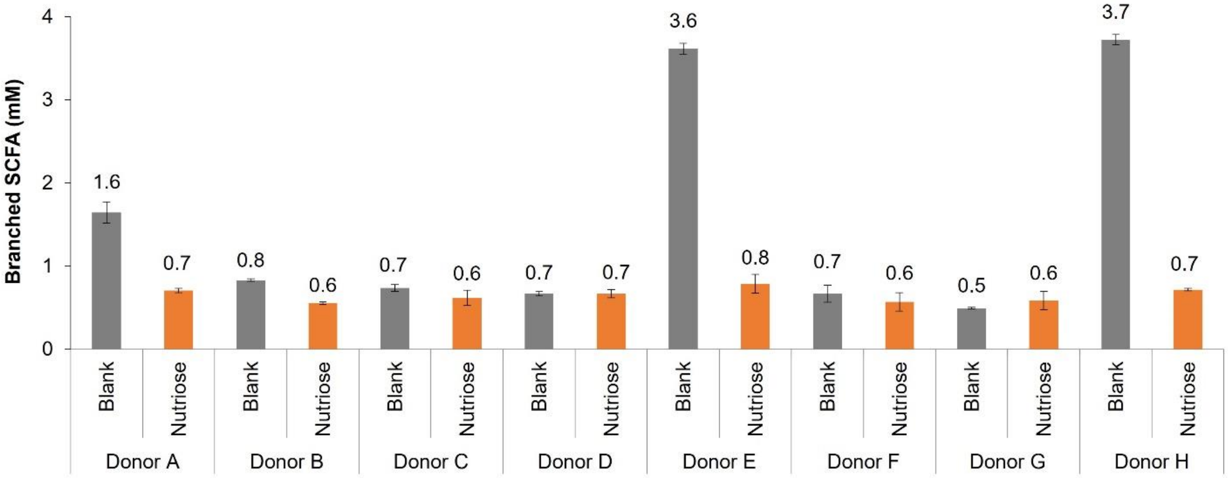
Branched SCFA (mM)
4
3
2
1
0
1.6
0.7
0.8
0.6
0.7
0.6
0.7
0.7
3.6
0.8
0.7
0.6
0.5
0.6
3.7
0.7
Blank
Nutriose
Donor A
Donor B
Donor C
Donor D
Donor E
Donor F
Donor G
Donor H

(f)

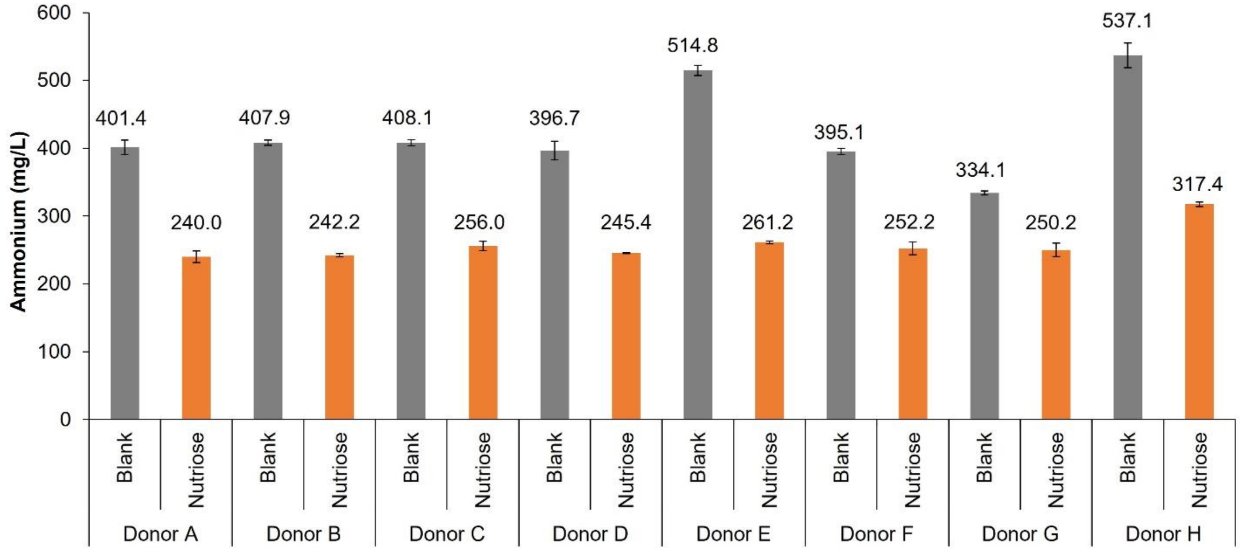
Ammonium (mg/L)
600
500
400
300
200
100
0
401.4
240.0
407.9
242.2
408.1
256.0
396.7
245.4
514.8
261.2
395.1
252.2
334.1
250.2
537.1
317.4
Blank
Nutriose
Donor A
Donor B
Donor C
Donor D
Donor E
Donor F
Donor G
Donor H
